# PSMA-positive prostatic volume prediction with deep learning based on T2-weighted MRI

**DOI:** 10.1007/s11547-024-01820-z

**Published:** 2024-05-03

**Authors:** Riccardo Laudicella, Albert Comelli, Moritz Schwyzer, Alessandro Stefano, Ender Konukoglu, Michael Messerli, Sergio Baldari, Daniel Eberli, Irene A. Burger

**Affiliations:** 1 Department of Nuclear Medicine, University Hospital Zürich, University of Zurich, Zurich, Switzerland; 2 Nuclear Medicine Unit, Department of Biomedical and Dental Sciences and Morpho-Functional Imaging, University of Messina, Messina, Italy; 3 Ri.MED Foundation, Palermo, Italy; 4 Institute of Diagnostic and Interventional Radiology, University Hospital Zurich, Zurich, Switzerland; 5 Institute of Molecular Bioimaging and Physiology, National Research Council (IBFM-CNR), Cefalù, Italy; 6 Computer Vision Lab, ETH Zurich, Zurich, Switzerland; 7 Department of Urology, University Hospital of Zürich, Zurich, Switzerland; 8 Department of Nuclear Medicine, Cantonal Hospital Baden, Baden, Switzerland

**Keywords:** Artificial intelligence, Neural network, PET/MRI, Prediction, Prostate cancer

## Abstract

**Purpose:**

High PSMA expression might be correlated with structural characteristics such as growth patterns on histopathology, not recognized by the human eye on MRI images. Deep structural image analysis might be able to detect such differences and therefore predict if a lesion would be PSMA positive. Therefore, we aimed to train a neural network based on PSMA PET/MRI scans to predict increased prostatic PSMA uptake based on the axial T2-weighted sequence alone.

**Material and methods:**

All patients undergoing simultaneous PSMA PET/MRI for PCa staging or biopsy guidance between April 2016 and December 2020 at our institution were selected. To increase the specificity of our model, the prostatic beds on PSMA PET scans were dichotomized in positive and negative regions using an SUV threshold greater than 4 to generate a PSMA PET map. Then, a C-ENet was trained on the T2 images of the training cohort to generate a predictive prostatic PSMA PET map.

**Results:**

One hundred and fifty-four PSMA PET/MRI scans were available (133 [^68^Ga]Ga-PSMA-11 and 21 [^18^F]PSMA-1007). Significant cancer was present in 127 of them. The whole dataset was divided into a training cohort (*n* = 124) and a test cohort (*n* = 30). The C-ENet was able to predict the PSMA PET map with a dice similarity coefficient of 69.5 ± 15.6%.

**Conclusion:**

Increased prostatic PSMA uptake on PET might be estimated based on T2 MRI alone. Further investigation with larger cohorts and external validation is needed to assess whether PSMA uptake can be predicted accurately enough to help in the interpretation of mpMRI.

**Supplementary Information:**

The online version contains supplementary material available at 10.1007/s11547-024-01820-z.

## Introduction

The use of multiparametric magnetic resonance imaging (mpMRI) and targeted biopsies in patients with elevated prostate-specific antigen (PSA) lead to a significant improvement in the detection of early prostate cancer (PCa) as has been shown in the PROMIS trial [1]. Using a standardized reporting system, called the Prostate Imaging Reporting and Data System (PIRADS), a sensitivity for significant PCa of 75–85% can be obtained [2], reaching 96% if combined with systematic biopsy. However, the probability to find significant PCa in PIRADS 3 lesions is only around 11.5% (4.35–18.7) [3]. Therefore, a large proportion of mpMRI-guided biopsies do not show any significant disease. Given that up to 20% of patients will have complications from targeted with systematic biopsies, improved sensitivity and especially specificity for the detection of PCa would be desirable.

Indeed, the prostate-specific membrane antigen (PSMA) targeted PET as a relatively new imaging modality was able to improve PCa assessment, from diagnosis to restaging [4–6]. Simultaneous PSMA PET/MRI further gave us the unique opportunity to directly assess the added value of PSMA PET to mpMRI. Accordingly, several studies suggested an increase in detection accuracy for significant PCa, if PSMA PET data are combined with the morphological information from T2 sequences compared to mpMRI alone [7–10]. However, PSMA is approved only for staging high-risk prostate cancer and detection of biochemical recurrence. Applying PSMA PET in all patients with suspected PCa would increase the costs significantly.

Recent observations have shown that high PSMA expression might be correlated with structural characteristics such as growth patterns on histopathology [11]. Such structural differences in histopathology cannot be recognized by the human eye on MRI images. However, deep structural image analysis using machine learning (ML) approaches might be able to detect such differences and therefore predict if a lesion would be PSMA positive just based on morphologic information. ML approaches have been used to predict molecular profiles of PCa [12], were used for lesion characterization with disease outcome prediction [13], and survival assessment [14]. These novel applications of machine-based learning on simultaneous PSMA PET/MRI may further improve image interpretation for PCa: from segmentation to accurate lesion analysis.

Therefore, we aimed to train a deep neural network (DNN) based on PSMA PET/MRI scans to predict increased prostatic PSMA uptake based on the axial T2 weighted sequence alone.

## Methods

### Patients

**Fig. 1:**
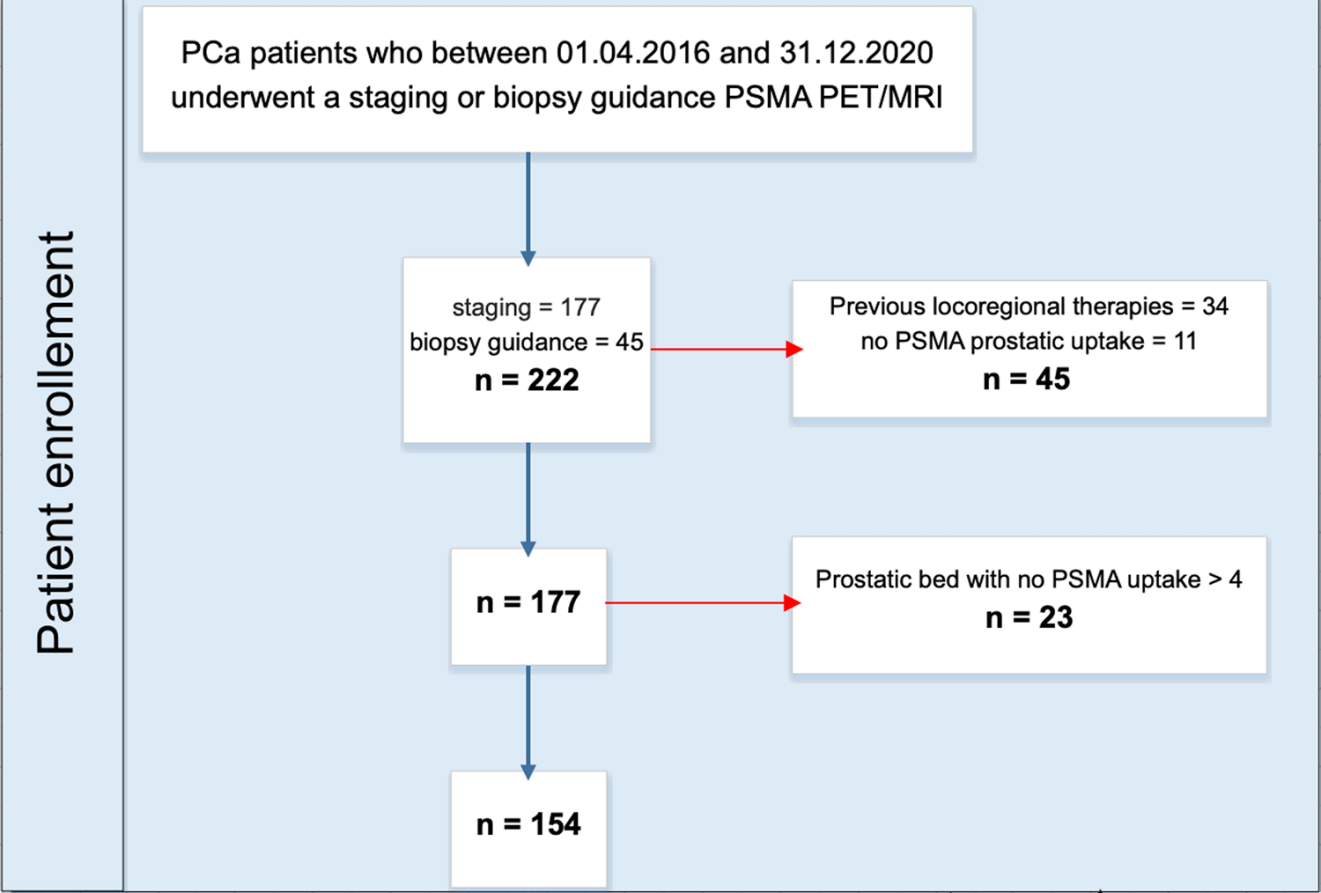
Patients’ enrollment flowchart. *PSMA* prostate-specific membrane antigen, *PET/MRI* positron emission tomography/magnetic resonance imaging

In this retrospective study, we screened all PCa patients who, between April 2016 and December 2020, consecutively underwent staging (*n* = 177) or prospectively biopsy guidance for PCa (*n* = 45) PSMA PET/MRI at our institution. Then, we excluded patients who previously underwent locoregional therapies (*n* = 34) or without any PSMA prostatic uptake (*n* = 11). Given the limited sample size, we decided to use every slice incorporating prostatic tissue on MRI and dichotomized the PSMA-uptake at a cut-off of SUV 4. This cutoff is based on suggestions that SUV 4 could be used for the differentiation of PCa with respect to normal prostatic gland uptake [15]. To further increase the specificity of our model, we excluded patients without PSMA uptake greater than 4 in the prostate (*n* = 23). In Fig. 1, we resumed the patients’ enrollment flowchart. The study was approved by the institutional review board (2020–02861); all staging patients signed a general informed consent for retrospective studies, while biopsy guidance patients signed the specific written informed consent of the prospective study [9].

### PSMA PET/MRI

All patients underwent [^68^Ga]Ga-PSMA-11 or [^18^F]PSMA-1007 PET/MRI scans (SIGNA PET/3T MRI, GE Healthcare, Waukesha, WI, USA). Images were acquired 60 and 90 min after the injection of [^68^Ga]Ga-PSMA-11 or [^18^F]PSMA-1007, respectively, starting with a whole-body MRI localizer scan. Then, a 3D dual-echo, spoiled gradient recalled echo sequence (LAVA-FLEX) for attenuation correction, and a PET emission scan were acquired. The protocol included dedicated sequences covering the pelvis, including a high-resolution T1-weighted LAVA-FLEX sequence, a T2-weighted fast recovery fast spin-echo sequence (FRFSE) in two planes, and diffusion-weighted images (DWI, with *b* values of 0, 400, and 700). Specifically, the axial T2 FRFSE were characterized by a repetition time (TR) of 2600 ms, an echo time (TE) of 117 ms, a flip angle (FA) of 125°, an acquisition matrix of 416 × 224 with a voxel size of 512 × 512, a slice thickness of 4 mm, a signal averages of 2, a bandwidth of 326 Hz/pixel, and an acquisition time of 3:48 mm:ss. Details of the other MRI sequences are given in supplemental Table 1. PET acquisition scan was acquired in the 3D time of flight (TOF) mode with a default number of 6-bed positions with an acquisition time per bed of 2 min (axial FOV of 25 cm and overlap of 24%, matrix of 256 × 256, 2 iterations, 28 subsets, with the sharpIR algorithm—GE Healthcare—and 5-mm filter cutoff). To reduce the radiopharmaceutical activity in the urinary system of [^68^Ga]Ga-PSMA-11, furosemide was injected intravenously 30 min before the tracer injection (0.13 mg/kg), and the patients were asked to void before the scan. All institutional protocols agreed with the joint EANM-SNMMI procedure guidelines [16].

### Image segmentation

For each anonymized patient, a nuclear medicine physician with 3 years of experience (R.L.) segmented the T2 MRI images of the whole prostatic gland using 3D Slicer software [17] version 4.11 through a free-hand, slice-by-slice segmentation on axial view. Those volumes were then confirmed by a double board-certified radiologist and nuclear medicine physician with 15 years of experience (I.A.B.). Then, the selected volumes of interest (VOIs) from MRI were transferred to the PSMA PET scans and dichotomized with a threshold at SUV 4 using the image biomarker standardization initiative (IBSI) compliant software Lifex [18] version 7.1 to generate positive and negative voxels (namely, binary masks: 1 for the target, 0 for the background).

Given that T2 and diffusion-weighted (DWI) MRI are the most important sequences always acquired to detect cancer, and that DWI is often limited in correlation due to distortions, we decided to use T2 weighted as the base from the mpMRI. Indeed, T2 images have a higher resolution compared to dynamic contrast-enhanced (DCE) or DWI (including apparent diffusion coefficient—ADC); we therefore consider that T2 images include a lot of “invisible” data that could be used by the neural network to predict the PSMA prostatic uptake.

### Deep learning

The customized-efficient neural network (C-ENet) [19] was used to predict increased prostatic PSMA uptake based on the axial T2 weighted sequence alone. ENet is a commonly used network in mobile applications where hardware availability is limited, and accurate segmentation is very critical [20]. Successively, it has been modified into C-ENet to be used in biomedical imaging applications, such as for segmentation of the prostate in MRI images [21]. Specifically, ENet is based on building blocks of residual networks, with each block consisting of three convolutional layers. It is characterized by asymmetric and separable convolutions with sequences of 5 × 1 and 1 × 5. The 5 × 5 convolution has 25 parameters while the corresponding asymmetric convolution has 10 parameters to reduce the size of the network. As explained in [19], the customization of ENet (namely, C-ENet) is achieved by replacing the output of 128 × 64 × 64 with an output of 256 × 64 × 64. This modification implies a dice similarity coefficient (DSC) distribution that, on average, is greater and exhibits less variability than the standard ENet. In our study, we used a C-ENet to automatically segment the prostate volume in the T2 MRI images and to automatically generate, within the obtained volume, a predictive PSMA PET map. To achieve these results, the C-ENet was trained twice. For the first training phase, the manual segmentations performed in the MRI dataset were used to automatically identify the anatomical prostate region. The stratified fivefold cross-validation strategy was used: in the way described in [22], the data set was divided into 5 folds of similar size, and the model was trained on five models by combining four of the five folds into a training set and keeping the remaining fold as validation. For the training task, an initial set of 16 patients was used to experimentally determine the best learning rate of 0.0001 with Adam optimizer [23]. Since the prostate segmentation suffers from unbalanced data (the prostate region is very small compared to the background), the Tversky loss function was used as loss function to adjust the weights of false positives and false negatives as reported in [24]. An automatic stop criterion was implemented in case the loss had not decreased for 10 consecutive epochs. Finally, we compared the proposed C-ENet method with the most widely used DL algorithm in biomedical image segmentation, namely UNet [25]. The distinctive feature of UNet is a U-shaped structure, where the network contracts the input image through a series of convolutional and pooling layers to capture context and then expands the representation back to the original input size to generate a segmentation map. The contracting path consists of convolutional layers followed by max-pooling layers to reduce spatial dimensions, while the expanding path involves up-sampling and concatenation operations to recover spatial information. Skip connections between corresponding layers in the contracting and expanding paths facilitate the retention of fine-grained details during segmentation.

**Fig. 2:**
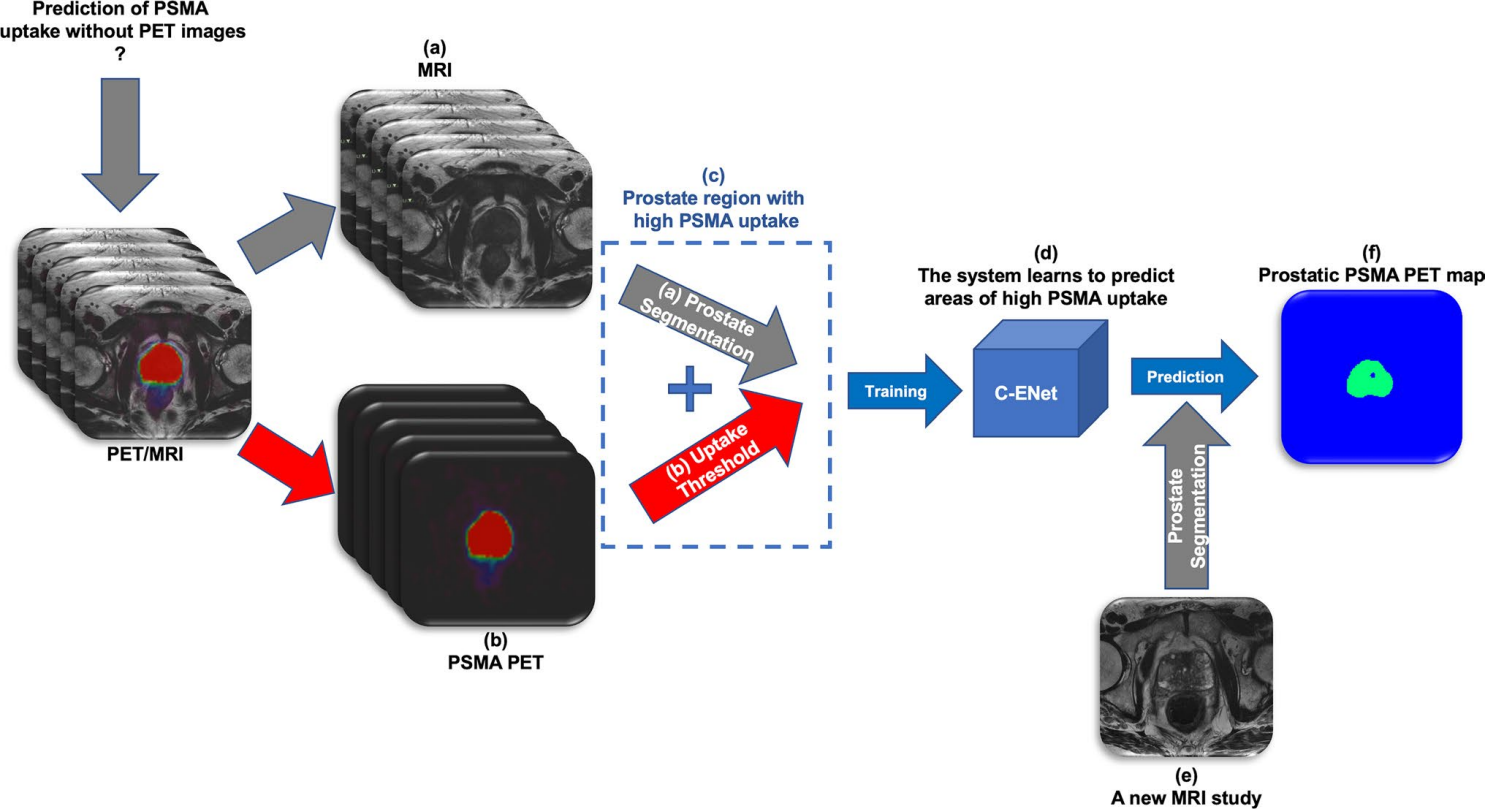
Flowchart for PSMA prostatic uptake prediction based on T2 MRI. Starting from the superimposition of the prostate region segmentation based on MRI images (**a**) and the binary PSMA uptake mask based on PET images (**b**), the prostate regions with high PSMA uptake (**c**) were used to train the C-ENet-based model (**d**). In this way, the model was able to predict prostatic PSMA PET maps (**f**) using the MRI dataset alone (**e**). *C-ENet* customized-efficient neural network, *MRI* magnetic resonance imaging, *PET* positron emission tomography, *PSMA* prostate-specific membrane antigen

For the second training phase, the prostate regions segmented in MRI images in the previous phase were superimposed on the binary PET masks obtained using LifeX. In this way, only the prostate regions with high PSMA uptake were considered in the MRI images given that the voxels belonging to the background (namely, the prostate region with low PSMA uptake) were set to zero value. Then, these images were used to train a new C-ENet-based model to generate a prostatic PSMA predictive PET map using the MRI dataset alone. Patients were divided into a training cohort (*n* = 124) and a validation cohort (*n* = 30) according to the general 80:20 rule, also known as the Pareto Principle. Specifically, the smaller lesions were used as a training set (lesion volume lower than 15 cc), while the larger ones were used as the test set to consider the problem of the partial volume effect in PET images [26] improving the robustness of this preliminary model. For preprocessing, MRI exams were resampled to an isotropic voxel size (1 × 1 × 1 mm^3^) with a matrix resolution of 512 × 512 using linear interpolation. Manually segmented masks were used as the ground truth (whole-gland) and PSMA prostatic uptake segmented masks were resampled using nearest-neighbor interpolation. For both models, data augmentation based on six different types of techniques (rotation, translation in both x and y directions, applying trimming, horizontal flipping, and zooming of the original images) was used to reduce overfitting. Finally, data standardization and normalization were used for faster convergence and to avoid numerical instability. The NVIDIA RTX A5000, 16 GB VRAM, 6144 CUDA Cores was used as a graphic processing unit (GPU). The flowchart of the proposed model is presented in Fig. 2.

### Statistical analysis

Statistical analyses were performed using SPSS statistics software, version 26 (IBM). Descriptive analyses were used to display patient data as means with standard deviations or medians with ranges to describe normally or non-normally distributed values, respectively; frequency distribution with percentages was used to summarize categorical variables. These analyses were performed by R.L. (MD, PhD). To provide a model evaluation, for each clinical case, we computed a set of performance indicators routinely used in the literature for shape comparison, namely sensitivity, positive predictive value (PPV), DSC, volume overlap error (VOE), and volumetric difference (VD):

$$\begin{aligned} & {\text{Sensitivity }} = {\text{ TP }}/{\text{ (TN }} + {\text{ FN)}} \\ & {\text{PPV }} = {\text{ TP }}/{\text{ (TP }} + {\text{ FN)}} \\ & {\text{DSC }} = { (}2 \, \times {\text{ TP) }}/{ (}2 \, \times {\text{ TP }} + {\text{ FP }} + {\text{ FN)}} \\ & {\text{VOE }} = \, 1 \, {-}{\text{ TP }}/{\text{ (TP }} + {\text{ FP }} + {\text{ FN)}} \\ & {\text{VD }} = \, \left| {{\text{FN}} - {\text{FP}}} \right| \, /{ (}2 \, \times {\text{ TP }} + {\text{ FP }} + {\text{ FN)}} \\ \end{aligned}$$

where TP, FP, TN, and FN are the true positives, false positives, true negatives, and false negatives, respectively. To verify if statistically significant different performances occurred between the two DL algorithms (i.e., C-ENet and UNet), the analysis of variance (one-way ANOVA) on the DSC was used. A *p* value < 0.05 was considered for statistical significance.

Statistical analyses for the model estimation were performed by A.C. (PhD).

## Results

One hundred fifty-four (154) patients (mean age 66.3 ± 7.75 years) with a median PSA of 11 ng/ml [2.5–480] underwent [^68^Ga]Ga-PSMA-11 (133/154) or [^18^F]PSMA-1007 (21/154) PET/MRI with an administered median dose of 124 MBq [76–198] or 245 MBq [135–303], respectively. Clinically significant PCa (ISUP ≥ 2) at biopsy were present in 127/154 of them (82.5%). Patients’ main characteristics are described in Table 1.

**Table 1:** Patients’ main characteristics

Patients’ number	154
[^68^Ga]Ga-PSMA-11 – [^18^F]PSMA-1007	133 (86.4%) – 21 (13.6%)
Median [^68^Ga] – [^18^F]PSMA dose	124 MBq [76–198] – 245 MBq [135–303]
Mean age at scan ± SD	66.3 ± 7.75 years
Median PSA at first scan*	11 ng/ml [2.5–480]
*Stage of disease*	
Staging	124 (80.5%)
Biopsy guidance	30 (19.5%)
*ISUP biopsy distribution*	
NA	11 (7.2%)
No PCa	6 (3.9%)
1	10 (6.5%)
2	21 (13.6%)
3	26 (16.9%)
4	47 (30.5%)
5	33 (21.4%)

*ISUP* international society of urological pathology, *NA* not available, *PCa* prostate cancer, *PSA* prostate-specific antigen, *PSMA* prostate specific membrane-antigen, *SD* standard deviation

*Missing 5 values

### MRI automatic prostate segmentation

**Fig. 3:**
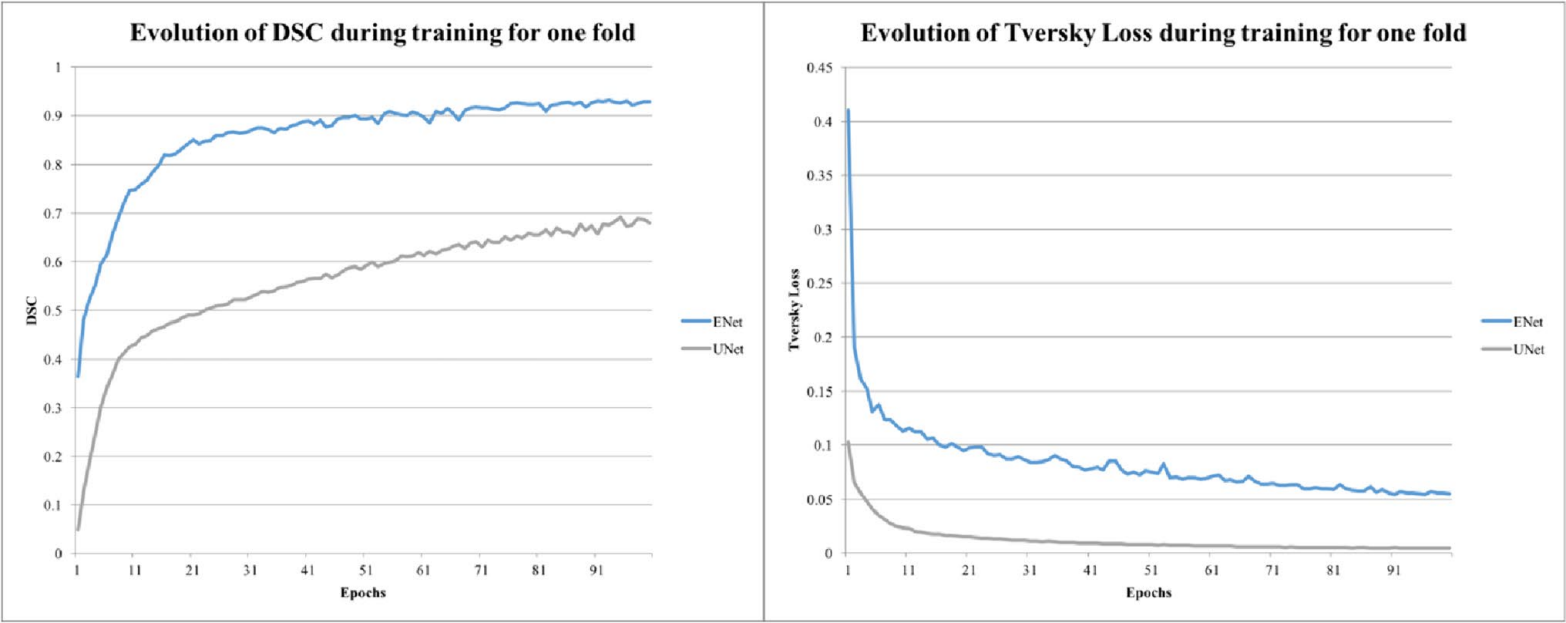
Training DSC and Tversky loss plots for C-ENet and UNet. *C-ENet* customized-efficient neural network, *DSC* dice similarity coefficient, *UNet* U-shaped neural network

For the automatic prostate segmentation from MRI images, we compared the proposed C-ENet method with UNet. Table 2 illustrates the performance metrics obtained by comparing the automatic (namely, C-ENet and U-shaped neural network—UNet) and manual delineations by averaging the results of the five validation folds during the five-fold cross-validation process reaching a DSC of 87.3%, which indicates excellent performances for C-ENet. Figure 3 shows the training DSC and the one-fold loss function plots: while the DSC in the training phase continues to increase over time reaching a DSC greater than 80% in just 15 iterations for C-ENet, the loss metric exhibits the opposite trend (a decrease over time) successfully reaching stability after more than 90 iterations.

**Table 2:** Performance results using C-ENet and UNet methods for prostate segmentation in MRI images

	Sensitivity (%)	PPV (%)	DSC (%)	VOE (%)	VD (%)
*C-ENet*					
Mean	90.8	84.8	87.3	22.3	8.2
± SD	5.6	7.3	3.8	5.8	15
± CI (95%)	1.1	1.45	0.8	1.15	3
*UNet*					
Mean	81.55	85.2	82.8	28.9	-3.3
± SD	9	7.3	6.15	8.2	15.4
± CI (95%)	1.8	1.4	1.2	1.6	3.05

*C-ENet* customized-efficient neural network, *DSC* dice similarity coefficient, *PPV* positive predictive value, *UNet* U-shaped neural network, *VD* volumetric difference, *VOE* volume overlap error

**Fig. 4:**
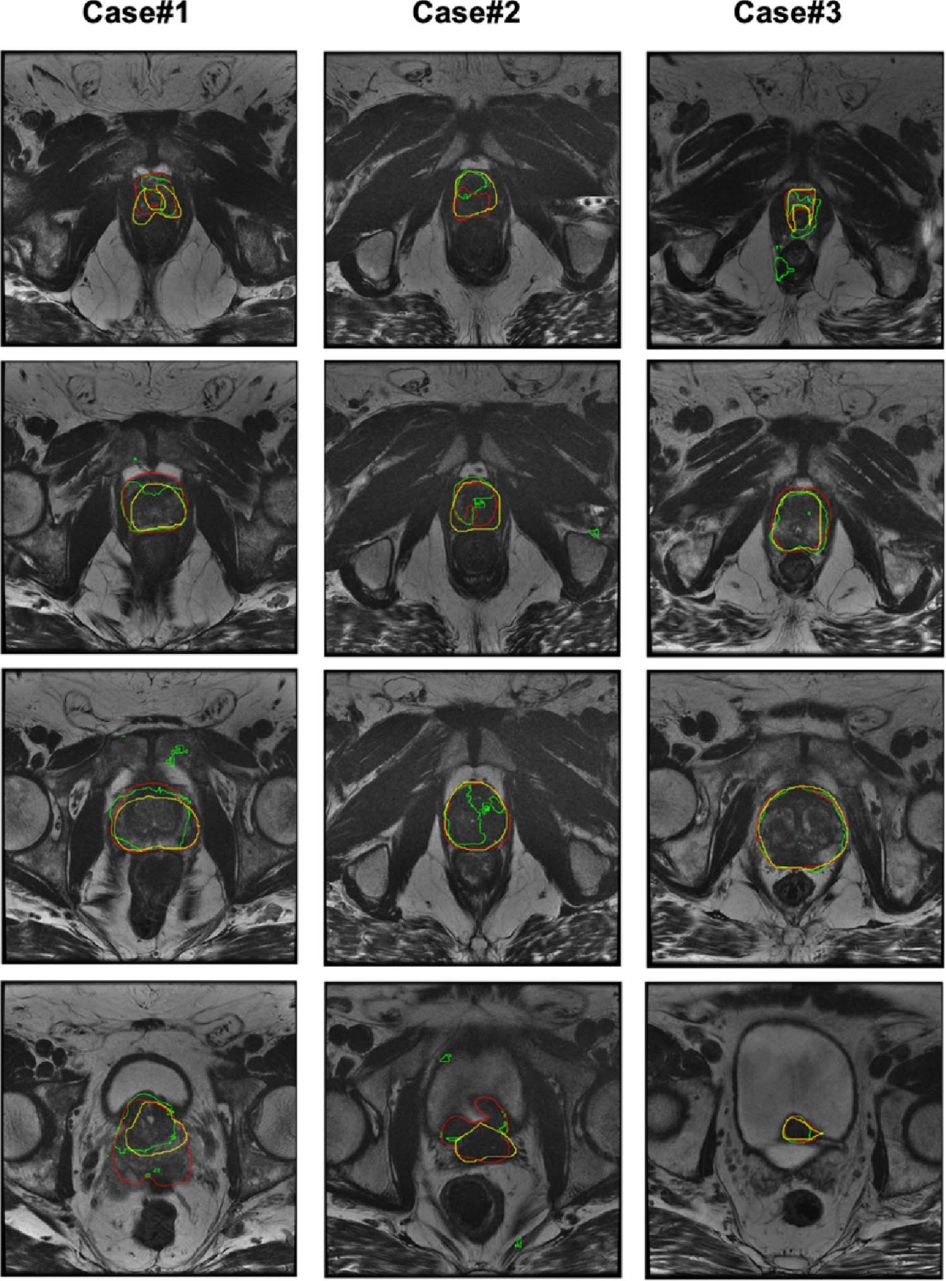
Comparison of MRI prostate gland segmentation performances for the two DL architectures in three patients (four different slices for each). The yellow contour indicates the gold standard (MRI prostate segmentation); the green contour indicates the UNet; the red contour indicates the C-ENet. For UNet, an average DSC of 77.3%, 48.8%, and 89.8% was obtained for the three cases, respectively. C-ENet performed better in each case with an average DSC of 79.95%, 85.6%, and 92.6%, respectively. *C-ENet* customized-efficient neural network, *DL* deep-learning, *DSC* dice similarity coefficient, *MRI* magnetic resonance imaging, *UNet* U-shaped neural network

ANOVA on the DSC showed a statistically significant difference among the volumes obtained using UNet and C-ENet (*F* value = 358.211, F-critic value = 3.889, *p* value = 1.11E-16). Therefore, the proposed C-ENet method outperformed the UNet. Finally, Fig. 4 shows the qualitative comparison between the two DL algorithms in three patients.

### PSMA prostatic uptake prediction based on T2 MRI

**Fig. 5:**
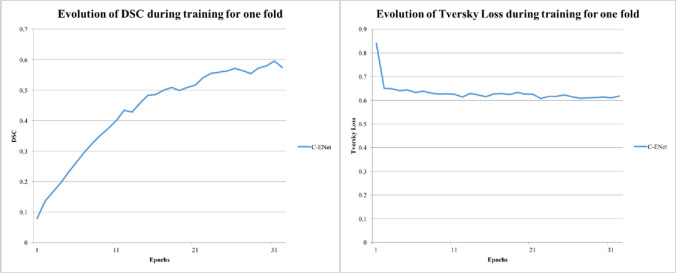
Training DSC and Tversky loss plots for C-ENet on prediction of PSMA prostatic uptake based on T2 MRI. *C-ENet* customized-efficient neural network, *DSC* dice similarity coefficient, *MRI* magnetic resonance imaging, *PSMA* prostate-specific membrane antigen

**Fig. 6:**
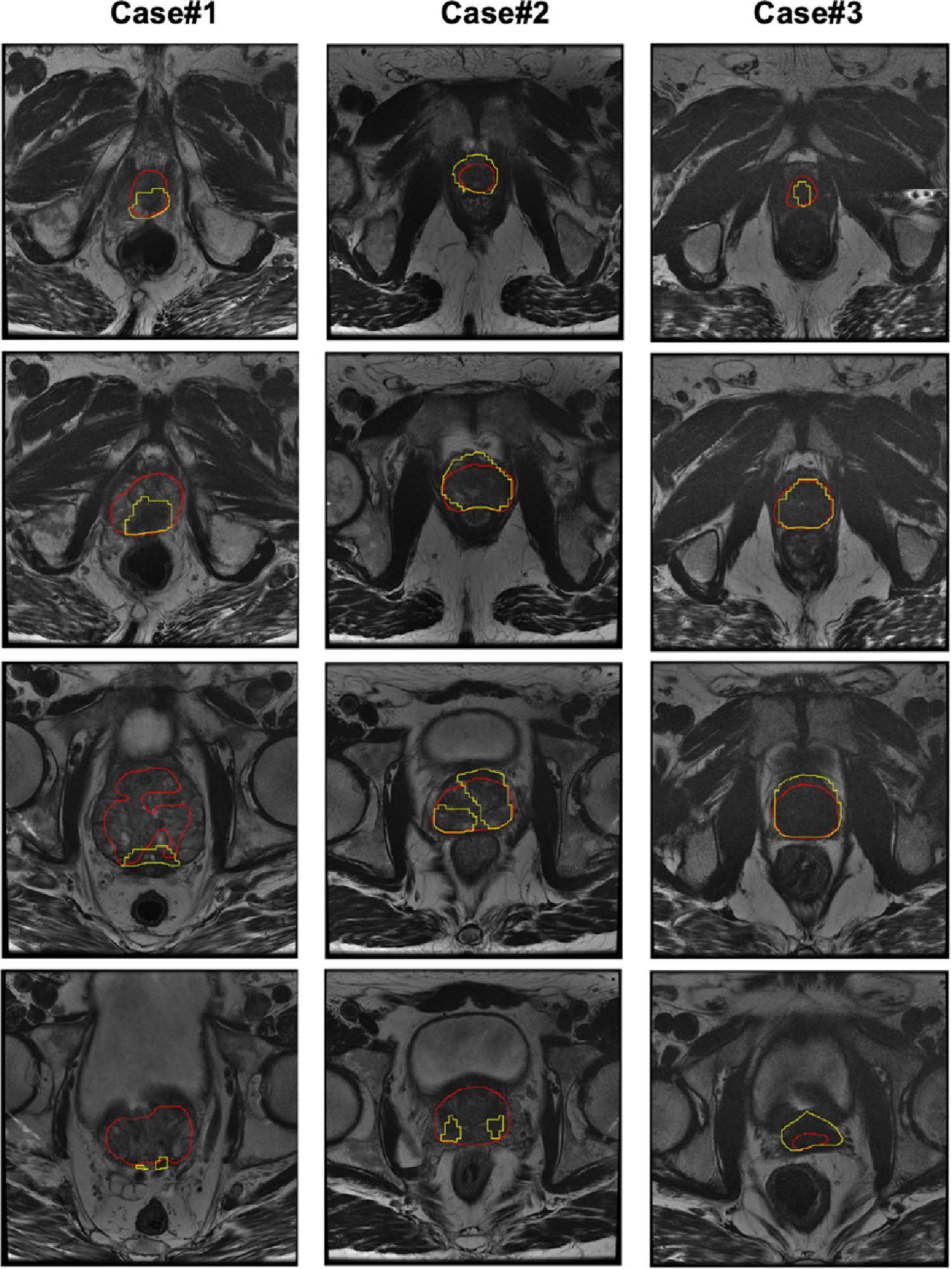
Qualitative comparison between the PSMA prostatic uptake based on T2 MRI (yellow contour) and C-ENet prediction (red contour) in three patients from the testing cohort: the less precise case (case #1, DSC = 20.6%), a case with heterogeneous uptake (case #2, DSC = 75.5%), and the more precise case (case #3, DSC = 85.9%). *C-ENet* customized-efficient neural network, *DSC* dice similarity coefficient, *MRI* magnetic resonance imaging, *PSMA* prostate-specific membrane antigen

For the second training phase, the segmented prostate volume was overlaid on the binary PET masks obtained using LifeX as described in the “Image Segmentation” section. In this way, only the prostate region with high PSMA uptake was considered in the MRI images given that the voxels belonging to the background (namely, the prostate region with PSMA uptake < 4) were set to zero value. These images were used to train a new C-ENet to generate a prostatic PSMA predictive PET map using the MRI dataset alone. Performance on the validation cohort showed a DSC of 69.5 ± 15.6%. All results are reported in Table 3. Figure 5 shows the training DSC and the loss function plots. Finally, Fig. 6 shows the qualitative comparison between the two DL algorithms in three patients.

**Table 3:** Performance results on the testing set using the C-ENet segmentation method for the prediction of PSMA prostatic uptake based on T2 MRI

	Sensitivity (%)	PPV (%)	DSC (%)	VOE (%)	VD (%)
Mean	77.6	67.1	69.5	44.9	39.05
± SD	10.55	22.9	15.6	16.2	78.7
± CI (95%)	3.8	8.2	5.6	5.8	28.2

*C-ENet* customized-efficient neural network, *DSC* dice similarity coefficient, *PPV* positive predictive value, *UNet* U-shaped neural network, *VD* volumetric difference, *VOE* volume overlap error

## Discussion

AI can magnify molecular imaging also with sustainable approaches to reduce potential gaps between hospitals with different facilities and among nations with different regulations. The worldwide access for patients with suspected PCa to pelvic MRI has significantly increased. Offering the same for PSMA PET, which has the potential to improve PCa detection would increase costs significantly. Here, we demonstrated the feasibility to predict prostatic PSMA uptake through a C-ENet model based only on T2-MRI images acquired from simultaneous PET/MRI, with a resulting DSC of 69.5 ± 15.6%, VOE of 44.9 ± 16.2%, and VD of 39.05 ± 78.7% in a homogeneous cohort of staging/biopsy guidance PCa patients naïve from any treatment. Only a few initial experiences using AI to predict PET images based on morphological data only have been published so far. In a somehow similar way, Chandrashekar et al. demonstrated that simulated FDG PET images can be obtained from non-contrast computed tomography (CT). In 298 patients with head and neck squamous cell carcinoma (HNSCC), they applied a random forest (RF) classification of CT-derived radiomic features to differentiate areas with negligible, low, and elevated FDG uptake. In the second step, a DL generative adversarial network (GAN) was trained for the CT to PET transformation task. Accordingly, the authors reported that tumors’ volumes were similar between the generated and ground-truth PET images concluding that the presented model resulted able to predict the clinical outcome with the same accuracy as that achieved using FDG-PET images without the need for tracer injection [27].

Also, Komori et al. aimed to develop an image-based DL technique able to generate delayed (60′) uptake patterns of [^11^C]PiB PET using only early-phase images obtained from 0 to 20 min after radiotracer injection, aiming to reduce the scan wait of patients with suspected Alzheimer disease. In a 253-patient cohort, the authors trained a neural network using early images as the input, and the corresponding delayed image as the output. A UNet CNN and a conditional-GAN (C-GAN) were used for the DL architecture and the data augmentation methods, respectively. As result, the concordance of amyloid positivity between the actual versus AI-predicted delayed images was 79% with a structural similarity index (SSIM) of 0.45 ± 0.04 [28].

In another setting with a smaller cohort including 64 patients with oropharyngeal cancer, Ji et al. described a CNN able to predict post-intensity-modulated radiotherapy (IMRT) [^18^F]FDG PET image. They used the pre-radiation [^18^F]FDG-PET image and the dose distribution map for the IMRT as the model’s inputs, while post-radiation [^18^F]FDG-PET images were the ground truth. Based on the Gamma test results, the achieved [^18^F]FDG-PET image prediction showed good agreement with the ground-truth images with similar SUV_mean_ values [29].

In the uncertain scenario of AI and a heterogeneous disease such as PCa [30], we selected a homogeneous cohort including only PSMA PET/MRI images of untreated patients. We consider the presented results as still suboptimal and not applicable for clinical use but given the existing limitations, a concordance of around 70% is satisfying for a preliminary experience. This should motivate to use future PSMA PET data sets for PCa detection together with mpMRI to further train and improve a predictive algorithm, that might be able to improve the interpretation of T2 weighted sequences and might serve as a potentially inexpensive method to better assess doubtful/unclear findings (i.e., PIRADS 3) determining if a patient requires further and more comprehensive examinations.

Limitations of this study include its retrospective nature, the use of a relatively small and monocentric patient cohort, and the missing external validation. Furthermore, it was not possible to incorporate DWI and ADC maps into the proposed model, due to image distortion and missing sequences for some patients. To overcome the potential difficulties due to the innate pathological heterogeneity in PCa, multicentric studies with a larger cohort and external validation, will hopefully also incorporate further mpMRI sequences which ultimately may improve the accuracy of our model.

## Conclusion

This study demonstrated the feasibility of an image-based DL prediction of prostatic PSMA PET uptake using only the T2-MRI images with a dice similarity of nearly 70%. Further investigation with larger cohorts and additional MRI sequences are needed to improve predicted PSMA uptake and evaluate if it might be good enough to improve the interpretation of mpMRI.

### Supplementary Information

Below is the link to the electronic supplementary material.

Supplementary file1 (DOCX 25 kb)
