# Correction: PSMA‑positive prostatic volume prediction with deep learning based on T2‑weighted MRI

**DOI:** 10.1007/s11547-024-01829-4

**Published:** 2024-08-24

**Authors:** Riccardo Laudicella, Albert Comelli, Moritz Schwyzer, Alessandro Stefano, Ender Konukoglu, Michael Messerli, Sergio Baldari, Daniel Eberli, Irene A. Burger

**Affiliations:** 1 Department of Nuclear Medicine, University Hospital Zürich, University of Zurich, Zurich, Switzerland; 2 Nuclear Medicine Unit, Department of Biomedical and Dental Sciences and Morpho-Functional Imaging, University of Messina, Messina, Italy; 3 Ri.MED Foundation, Palermo, Italy; 4 Institute of Diagnostic and Interventional Radiology, University Hospital Zurich, Zurich, Switzerland; 5 Institute of Molecular Bioimaging and Physiology, National Research Council (IBFM-CNR), Cefalù, Italy; 6 Computer Vision Lab, ETH Zurich, Zurich, Switzerland; 7 Department of Urology, University Hospital of Zürich, Zurich, Switzerland; 8 Department of Nuclear Medicine, Cantonal Hospital Baden, Baden, Switzerland

**Correction to: La Radiologia Medica** 10.1007/s11547-024-01820-z

In the original publication of the article, reference [9] was published incorrectly. The correct reference is given below:

9. Ferraro DA, Becker AS, Kranzbühler B, Mebert I, Baltensperger A, Zeimpekis KG, Grünig H, Messerli M, Rupp NJ, Rueschoff JH, Mortezavi A, Donati OF, Sapienza MT, Eberli D, Burger IA (2021) Diagnostic performance of ^68^Ga-PSMA-11 PET/MRI-guided biopsy in patients with suspected prostate cancer: a prospective single-center study. Eur J Nucl Med Mol Imaging 48(10):3315–3324. 10.1007/s00259-021-05261-y

The original article has been corrected.

