# Supplementary material for: PSMA-positive prostatic volume prediction with deep learning based on T2-weighted MRI
Source: Radiol Med. 2024 May 3;129(6):901–11. doi: 10.1007/s11547-024-01820-z (PMC11168990; doi:10.1007/s11547-024-01820-z)
Supplement: Supplementary file 1 — Supplementary file1 (DOCX 25 kb) [file 11547_2024_1820_MOESM1_ESM.docx]

**Supplement Table 1.** MRI sequences details

|  | Axial DWI EPI (Focus) (Pelvis) | Axial LAVA-FLEX WB (DIXON) | Axial T1w Whole ARC (Pelvis) | Axial T2w FRFSE-XL  (Pelvis) | Coronal T2w WB FRFSE-XL | Coronal T2w  FRFSE-XL  (Pelvis) | Axial DCE  (Lava Dyn)  (Pelvis) |
| --- | --- | --- | --- | --- | --- | --- | --- |
| Repetition time, TR (ms) | 4000 | 5.6 | 550 | 2600 | 6304 | 2900 | 6.361 |
|  |  |  |  |  |  |  |  |
| Echo time,  TE (ms) | 67.3 | 1.3-2.7 | 8.26 | 117 | 123 | 121 | 2.376 |
|  |  |  |  |  |  |  |  |
| Flip angle,  FA (degrees) | 90 | 12 | 111 | 125 | 111 | 125 | 30 |
|  |  |  |  |  |  |  |  |
| Acquisition matrix | 160 x 80 | 344 x 256 | 352 x 352 | 416 x 224 | 288 x 224 | 416 x 224 | 160 x 80 |
|  |  |  |  |  |  |  |  |
| Image size (voxels) | 256 x 256 | 512 x 512 | 512 x 512 | 512 x 512 | 512 x 512 | 512 x 512 | 288 x 192 |
|  |  |  |  |  |  |  |  |
|  |  |  |  |  |  |  |  |
| Slice thickness (mm) | 4 | 3 | 5 | 4 | 5 | 4 | 4 |
|  |  |  |  |  |  |  |  |
| Signal averages | 8 | 0.68 | 0.5 | 2 | 0.5 | 4 | 0.35 |
|  |  |  |  |  |  |  |  |
| b-values (s/mm2) and signal averages | 0 (6 av.)  400 (8 av.)  700 (16 av.) |  |  |  |  |  |  |
|  |  |  |  |  |  |  |  |
| Diffusion direction | ‘All’ |  |  |  |  |  |  |
|  |  |  |  |  |  |  |  |
| Bandwidth (Hz/pixel) | 1953 | 166 | 1953 | 326 | 355 | 326 | 62.5 |
|  |  |  |  |  |  |  |  |
| Acquisition time (mm:ss) | 5:41 | 0:18 | 3:53 | 3:48 | 0:24 | 4:04 | 3:27 |
